# Serological and molecular detection of pathogenic *Leptospira* in domestic and stray cats on Reunion Island, French Indies

**DOI:** 10.1017/S095026882100176X

**Published:** 2021-08-10

**Authors:** M. Holzapfel, F. Taraveau, Z. Djelouadji

**Affiliations:** USC1233-INRA, Rongeurs Sauvages, Risque Sanitaire et Gestion des Populations, Établissement VetAgro Sup, Campus vétérinaire de Lyon, Marcy l'Etoile, France

**Keywords:** Cat, epidemiology, leptospirosis, public health, reservoir

## Abstract

Indian Ocean islands are endemic areas for human and animal leptospirosis. Maintenance host species for *Leptospira* spp. have still not been completely elucidated, and recently the role of cats (*Felis catus*) has been questioned. This cross-sectional study aims to determine whether cats are part of the maintenance community of different strains of *Leptospira* spp. in Reunion Island. The prevalence of *Leptospira* infection in an opportunistic sample of stray and domestic cats (*n* = 92) from Reunion Island has been studied using serological (microagglutination test) and molecular detection (polymerase chain reaction (PCR)). The results revealed a seroprevalence of 37.0% (34/92) (cut-off 1:40) without a significant difference in the living conditions of animals. The predominant serogroup was Icterohaemorrhagiae, but Ballum, Cynopteri and Australis were also detected. Using PCR, 28.6% (12/42) of stray cats were tested positive. Leptospiral DNA was detected in renal tissue, urine and blood of respectively 14.3% (6/42), 10.3% (4/39) and 11.9% (5/42) of stray cats, but 0% (0/3), 0% (0/50) and 0% (0/36) of domestic cats (*P* = non-applicable, *P* = 0,038, *P* = 0,058 respectively). Partial *rrs* gene (16S rRNA) sequencing identified *Leptospira interrogans* in all PCR-positive samples. Our study confirms that renal carriage and urinary shedding are possible, positioning cats, and especially stray cats as potential actors within the maintenance community of *L. interrogans* in Reunion Island.

## Introduction

Leptospirosis is a worldwide zoonotic disease caused by spirochetes from pathogenic and intermediate *Leptospira* species. The human incidence is estimated at 1 million cases per year [1]. Leptospirosis is a re-emerging infectious disease, especially in tropical and subtropical regions, where environmental conditions favour the survival and transmission of leptospires [2].

The incidence ranges from 0.1 to 1 case/100 000 inhabitants per year in temperate areas to over 100 cases/100 000 inhabitants per year in the tropics [3]. In mainland France, the incidence reached 1 case/100 000 inhabitants in 2014, which represents 600 cases per year [4]. In Reunion Island, a French department located in the south-western Indian Ocean, the annual incidence ranged from 3 to 10 cases/100 000 inhabitants between 2004 and 2015 [5]. Consequently, leptospirosis is regarded as an endemic disease and a crucial public health issue on Reunion Island.

More than 150 mammalian species can harbour *Leptospira* spp. The rat (*Rattus rattus*) is considered as the main maintenance host, even though studies have shown that many other mammals, such as hedgehog and mustelid species, can carry pathogenic leptospires [6]. Maintenance hosts excrete leptospires and contaminate the hydric environment for long periods of time, independently of the presence of clinical signs. The maintenance hosts either develop chronic infection of the renal tubules while remaining asymptomatic, or present acute disease [7]. The infection of animals and humans occurs from direct contact with the urine of infected animals or indirectly from contaminated water [8].

Cats are susceptible to *Leptospira* spp., but the prevalence of clinical leptospirosis in this species is low [9]. Cats can present mild polyuria/polydipsia and increased body temperature after infection. Liver and kidney lesions have also been reported together with the presence of *Leptospira*-specific antibodies [10].

However, the role of cats in the epidemiology of leptospirosis remains poorly understood. Recent studies testing the seroprevalence of *Leptospira* spp. in cats in various geographic regions reported from 5% to 36% seropositivity [11–16]. Moreover, in the tropical island of Christmas Island, a study found a much higher prevalence of leptospiral DNA carriage in feral cats than in rats [12]. On Reunion Island, a seroprevalence of 26.7% (cut-off 1:100) and renal carriage of 28.6% were registered in stray cats (*n* = 30) [15]. Yet, a recent study on feral cats reported only one renal carriage out of 172 animals tested with quantitative real-time polymerase chain reaction (PCR) (0.6%). Interestingly, this isolated strain presented high DNA sequence similarity with a strain isolated from an acute human case of leptospirosis on the island [17].

Evidence has indicated that cats can intermittently shed leptospires in their urine up to 12 weeks following infection [13]. In recent studies, leptospires from cat urine samples were successfully grown in culture, emphasising the potential role of cats in the contamination of the environment and transmission to other species [18, 19].

The first aim of the present study was to estimate the seroprevalence for different serovars of *Leptospira* spp. among cats and to determine the main serogroups circulating in domestic and stray cats from Reunion Island. The second purpose was to record the urine excretion, kidney carriage and presence of *Leptospira* spp. in the blood to consider possible long-term carriage and shedding of the bacteria from this species; and to identify causal *Leptospira* strains by sequencing.

## Materials and methods

### Ethics statement

The present study was approved by the ethical committee of VetAgro Sup (named ethical committee Jacques Bonnod) under the licence number 1970-18. This committee is registered at the French Ministry of Research and reviews all license applications for preclinical research and all clinical research projects. The use of animals for scientific purposes is covered in France, by the European Directive 2010/63/EU [20], that was transposed into French law on 1 February 2013.

### Sample collection

Experiments were designed to estimate the apparent prevalence of *Leptospira* spp. in stray and domestic cats from northern and western Reunion Island. The sample size of this study was calculated to ensure estimation with a precision of at least 10% (i.e. obtaining a confidence interval (CI) of less than 20% width). Theoretical seroprevalence was fixed at 27% [15], confidence levels to 95% and the population of cats in the Reunion Island was considered as infinite regarded to the sample size of the study. The optimal sample size (*n* = 87) was determined with a binomial test. Finally, we included a total of 92 cats, presenting different living conditions (domestic *vs.* stray cats). Domestic cats (*n* = 50) were selected among voluntary clients from the Veterinary Clinic of Front-de-Mer in Saint-Paul. Domestic cats included in the study were healthy and were presented for sterilisation; animals under antibiotic treatments or presenting underlying diseases were excluded. All cats lived partially outdoor. Three of the domestic cats included in the study were euthanised by the veterinarians for owners’ personal reasons. Stray cats (*n* = 42) were captured in urban areas, by the animal shelters of Sainte-Marie and Saint-Paul. If neither identified nor adopted after 8 days, the cats were sedated using acepromazine and ketamine and euthanised by the shelter with pentobarbital after blood and urine were collected. Health check was not performed on stray animals, but at the time of necropsy, no macroscopic lesion compatible with leptospirosis was observed (jaundice, hepatomegaly and no visible renal changes) [10]. At the time of the consultation or necropsy, no domestic or stray cats respectively were excluded because all of them fit in the inclusion criteria. For each cat, 1–4 ml of blood was collected from the cephalic vein or jugular vein in a dry tube and in an ethylenediaminetetraacetic acid (EDTA) tube. During the hours following sampling, the dry tube was centrifuged for 15 min at 2000 ***g***, and the serum was separated. Both the serum and the EDTA tube were stored at 4 °C for further analyses. Urine samples were collected by cystocentesis (1–5 ml) and kept in a dry tube neutralised with 0.5 ml of phosphate-buffered saline (PBS) at 4 °C. Kidneys were collected from euthanised animals in a sterile manner, and renal tissue was stored in sterile tubes at 4 °C.

A total of 92 cats were sampled between 1 February and 27 February 2013 (rainy season). The samples included 92 blood (92 for serological analysis and 78 for PCR), 89 urine and 45 kidney samples. All samples were kept at +4 °C (maximum 7 days) and sent progressively to the Leptospires Laboratory at VetAgro Sup (the veterinarian school of Lyon, France) for serological and molecular analyses.

### Microscopic agglutination test (MAT)

Sera were tested against 24 pathogenic serovars of *Leptospira* spp. and two non-pathogenic serovars (Table 1), selected based on their prevalence in French overseas territories and on the reference strains available at the Leptospires Laboratory at VetAgro Sup [21]. Sera were centrifuged for 5 min at 3600 ***g***. Then, each well of a sterile microtitre plate was filled with 25 μl of serum in Sorensen buffer (1:20 dilution) and 25 μl of a specific leptospiral strain (serum diluted at 1:40 in the final solution). For each leptospiral strain, controls consisted of replacing the cat's serum with 25 μl of a serum with antibodies specific for the strain tested (positive control) or with 25 μl of Sorensen buffer (negative control).

**Table 1.** Leptospiral strains used as capture antigens for micro agglutination test

Serogroup	Serovar	Strain	Abbr.	Species
Australis	Australis	Ballico	AUS	*L. interrogans*
	Muenchen	Muenchen C90^a^	MUN	*L. interrogans*
	Bratislava	Jez Bratislava^a^	BRAT	*L. interrogans*
Autumnalis	Autumnalis	Akiyami A^a^	AKI	*L. interrogans*
	Bim	Strain 1051^a^	BIM	*L. kirschneri*
Ballum	Castellonis	Castellòn 3^a^	BAL	*L. borgpetersenii*
Bataviae	Bataviae	Van Tienen^a^	BAT	*L. interrogans*
Canicola	Canicola	Hond Utrecht IV	CAN	*L. interrogans*
Cynopteri	Cynopteri	Strain 3522C^a^	CYN	*L. kirschneri*
Grippotyphosa	Grippotyphosa	Moskva V^a^	GRIP	*L. kirschneri*
	Vanderhoedoni	Kipod 179^a^	VAN	*L. kirschneri*
Hebdomadis	Kremastos	Kremastos^a^	HEB	*L. interrogans*
Icterohaemorrhagiae	Icterohaemorrhagiae	Strain 19	19	*L. interrogans*
	Copenhageni	Strain M20	COP	*L. interrogans*
	Icterohaemorrhagiae	Verdun	VER	*L. interrogans*
Panama	Panama	Strain CZ 214K^a^	PAN	*L. noguchii*
Pomona	Pomona	Pomona^a^	POM	*L. interrogans*
Pyrogenes	Pyrogenes	Salinem^a^	PYR	*L. interrogans*
Sejroe	Sejroe	Strain M84^a^	SJ	*L. borgpetersenii*
	Saxkoebing	Strain Mus 24^a^	SAX	*L. interrogans*
	Hardjo	Hardjoprajitno	HJ	*L. interrogans*
	Wolfii	Strain 3705^a^	WOLF	*L. interrogans*
Tarassovi	Tarassovi	Perepelitsin^a^	TAR	*L. borgpetersenii*
Mini	Mini	Sari^a^	MINI	*L. borgpetersenii*
Semaranga	Patoc	Patoc 1^a^^,^^b^	Bifl P	*L. biflexa*
	Semaranga	Veldrat^a^^,^^b^	Meyeri	*L. meyeri*

Serogroup and serovar according to antigenic classification, complete name and abbreviation (Abbr.) of the strain used, and species according to genomic classification.

a Reference strain.

b Non-pathogenic *Leptospira* strain.

Microtitre plates were then incubated for 1 h at 37 °C followed by 1 h at 4 °C and examined using a dark-field microscope. All positive sera were tested again for the suspected serovars by using serial dilutions to get titres ranging from 1:20 to 1:320. The endpoint titre was considered the greatest dilution showing at least 50% agglutination compared to the control. Titres of 1:40 or higher were considered as indicators of exposure for the strain tested, as described in previous studies [22–24]. For sera reacting with more than one serovar, the seropositivity was attributed to the strains with the highest titre and serovars with lower agglutination titres were considered as cross-reactions.

### DNA extraction

DNA was extracted with a KingFisher^®^ automation instrument (Thermo Scientific, Illkirch, France) and a universal kit (LSI MagVet Universal Kit MV384, Thermofisher, Courtaboeuf, France) using 200 μl blood, pellet from centrifuged urine (1 ml of urine was centrifuged at 3000 ***g*** for 10 min at room temperature, pellet was washed with 1 ml PBS 1× and the pellet of a second centrifugation at 2000 ***g*** for 10 min was used) or 25 mg of minced renal tissue (cortico-medullary junction). PCR and real-time PCR (qPCR) were performed as described below in the Leptospires Laboratory at VetAgro Sup.

### Real-time PCR

Real-time PCR (qPCR) was conducted using the LSI kit (TaqVet PathoLept kit, Lifetech, Lissieu, France), which amplifies a specific region of pathogenic *Leptospira* spp. The qPCR were performed using 20 μl of mix and 5 μl of extracted DNA. Positive and negative controls were included by replacing 5 μl of sample DNA with 5 μl of *Leptospira interrogans* serogroup Icterohaemorrhagiae DNA or 5 μl of PBS, respectively. qPCR was carried out using Rotorgene 6000 machine (Qiagen, Courtaboeuf, France), samples with a cycle threshold (Ct) over 38 were considered negative (cycle threshold corresponds to the number of qPCR cycle from which an amplified DNA is detected by fluorescence).

### End-point PCR and sequencing

All positive samples analysed by qPCR were further submitted to species identification using the *rrs* (16S rRNA) sequencing. The extracted DNA (5 μl) was added to the amplification mix (45 μl; buffer 10×; MgCl_2_ 25 mm; dNTPs 10 mm each; primer F 10 μm; primer R 10 μm and TaqPolymerase 5 U/μl). The primers used targeted the 331-bp *rrs* gene with the following primer sequences: forward: 5′-GGCGGCGCGTCTTAAACATG-3′ and reverse: 5′-TTCCCCCCATTGAGCAAGATT-3′. Positive and negative controls were included by using 5 μl of *L. interrogans* serogroup Icterohaemorrhagiae DNA and 5 μl of PBS instead of sample DNA, respectively. The PCR cycling programme was 15 min at 95 °C, followed by 40 cycles of 30 s at 95 °C, 30 s at 58 °C and 1 min at 72 °C, followed by 10 min at 72 °C for final elongation. PCR was performed using Sure Cycler 8800 machine (Agilent Technologies, Les Ulis, France). The results were evaluated under UV light after migration using agarose gel electrophoresis. All PCR products were subjected to Sanger sequencing with the BigDye Terminator sequencing kit using a 3730XL DNA analyzer (Applied Biosystems, Saint Aubin, France). The *Leptospira* species were identified using the sequence information from NCBI nucleotide BLAST (http://blast.ncbi.nlm.nih.gov).

### Statistical analysis

CIs around proportion were calculated assuming a binomial distribution and calculated with the binom.test function of R (Version 4.0.3 (2020-10-10), R: A language and environment for statistical computing, R foundation for statistical computing, Vienna, Austria). The chi-squared test was used to compare seroprevalence and a Fisher exact test to compare DNA carriage between stray and domestic animals. Differences were considered statistically significant when *P* < 0.05. Due to the sampling size, cats were not classified into groups by age, sex and breed.

## Results

### Serological results

**Fig. 1.**
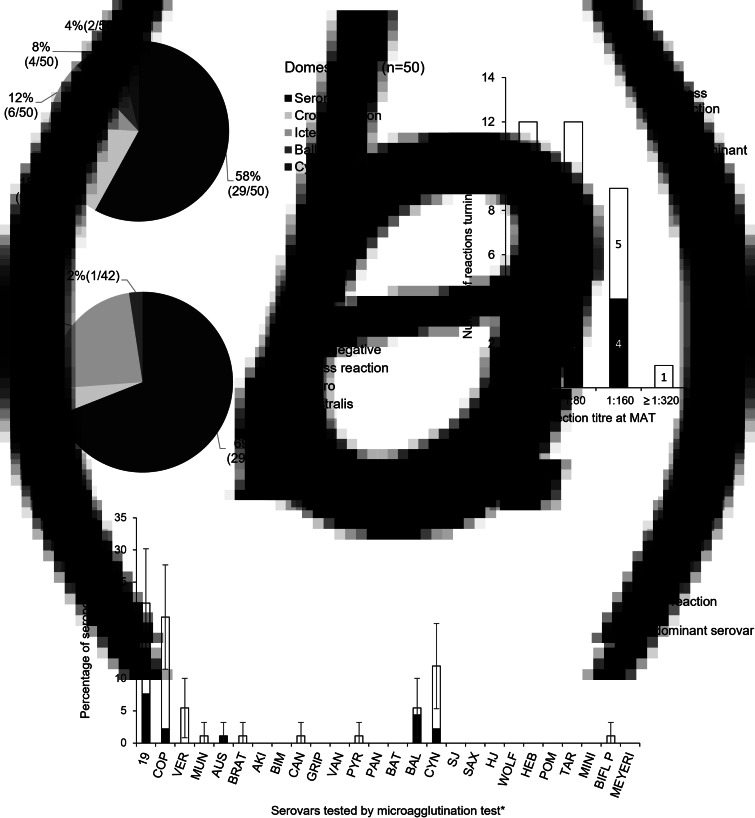
Seroprevalence of cats tested by micro-agglutination test (*n* = 92). (a) Percentage of animals tested negative or positive for one serogroup of leptospires or several serogroups (cross-reaction) via MAT according to their condition of living, stray cats (*n* = 42) or domestic cats (*n* = 50). (b) Higher dilution for which the MAT is positive (*n* = 34) for one predominant serovar and for any serovar. Cross-reactions (between two or more serovars) and reactions with one predominant serovar are presented. (c) Percentage of seropositive samples (*n* = 92) for each of the 26 *Leptospira* spp. serovars tested (*see Table 1 for abbreviations). Cross-reactions (between two or more serovars) and reactions with one predominant serovar are presented. CIs at 95% are indicated. MAT cut-off titre 1:40.

During February 2013, 92 sera samples were collected from 50 domestic cats and 42 stray cats and analysed by MAT. With a cut-off of 1:40, 34 sera were tested positive (37.0% with 95% CI (27.1–46.9)). The seroprevalence was estimated to be 42.0% (95% CI (28.3–55.6)) among domestic cats and 31.0% (95% CI (17.0–45.0)) among stray cats (Fig. 1a). No significant difference between the two groups was revealed (chi-squared test, *χ*^2^ (1, *N* = 92) = 0.77, *P* = 0.38).

The antibody titre ranged from 1:40 to 1:320 (Fig. 1b). Only one animal presented an antibody concentration high enough to be detected at a titre of 1:320, and 71% of positive results were obtained at dilutions of 1:80 or 1:40.

The positive MAT results revealed serogroup Icterohaemorrhagiae to be the main serogroup in circulation with 47% of the positive results. Considering serovars, the main serovars detected were Icterohaemorrhagiae Icterohaemorrhagiae (19 and VER) and Ballum Castellonis (BAL), with seroprevalences of 7.6% (7/92) and 4.3% (4/92), respectively, followed by serovars Cynopteri Cynopteri (CYN, 2/92), Icterohaemorrhagiae Copenhageni (COP, 2/92) and Australis Australis (AUS, 1/92) (Fig. 1c).

The results can also be considered using the serogroups from which the serovars belong. In this case, 22 cats were positive for only one serogroup (Icterohaemorrhagiae, Australis, Ballum or Cynopteri), one cat was positive for two different serogroups (Icterohaemorrhagiae and Cynopteri) with one predominant serogroup (Icterohaemorrhagiae) and 11 cats were presenting cross-reactions between two or more serogroups. Among domestic cats, specific antibodies for the serogroup Icterohaemorrhagiae were present in 12% (6/50) of the tested animals, while Ballum was detected in 8% (4/50) and Cynopteri was detected in 4% (2/50) of the tested animals. Among stray cats, two serogroups were detected: Icterohaemorrhagiae at 24% (10/42) and Australis at 2% (1/42).

A total of 53% (18/34) of the positive sera presented antibodies for several serovars. Including cross-reactions, three serogroups appeared to be more prevalent than the other serogroups: Icterohaemorrhagiae (19, COP and VER), Ballum (BAL) and Cynopteri (CYN) (Fig. 1c). Additionally, serovars Icterohaemorrhagiae and Copenhageni (12 animals out of 19 cross-reactions), as well as Copenhageni and Cynopteri (7/19) and Icterohaemorrhagiae and Cynopteri (5/19) often cross-reacted.

### Molecular results

Due to technical issues (insufficient volume of blood or urine, alteration of samples during transport), some samples could not be used for PCR analyses. A total of 78 blood samples and 89 urine samples were tested. Renal tissue samples were collected from euthanised animals only, which represented 45 cats.

Regardless of the template tested, 12 out of 92 animals (13% (6.1–19.9%)) were tested positive by PCR analyses, with Ct ranging between 30 and 34. More specifically, leptospiral DNA was found in 5/78 (6.4%) blood samples, 4/89 (4.5%) urine samples and 6/45 (13%) renal samples (Table 2). All positive results were detected in 12 stray cats (Table 3), with four of them harbouring leptospiral DNA only in their blood, two shedding leptospiral DNA only in urine and four harbouring leptospiral DNA only in kidneys. One animal presented both urinary and kidney carriage, and one animal tested positive in all three templates. No leptospiral DNA was detected in domestic cats. There was weakly statistically significant difference between stray and domestic cats in the proportion of PCR-positive urine samples (Fisher's exact test, *P* = 0.034) and PCR-positive blood samples (Fisher's exact test, *P* = 0.058), with stray cats more likely to present positive results.

**Table 2.** PCR results for blood, urine and renal samples of domestic and stray cats, and comparison with seroprevalences

Population considered	PCR on blood samples*	PCR on urine samples**	PCR on renal samples	Seroprevalence (MAT)
Stray cats (*n* = 42)	11.9% (5/42) [4.0–25.6]	10.3% (4/39) [2.9–24.2]	14.3% (6/42) [5.4–28.4]	31.0% (13/42) [17.6–47.1]
Domestic cats (*n* = 50)	0% (0/36) [0–9.7]	0% (0/50) [0–7.1]	0% (0/3) –	42.0% (21/50) [28.2–56.8]
All cats (*n* = 92)	6.4% (5/78) [2.1–14.3]	4.5% (4/89) [1.2–11.1]	13.3% (6/45) [5.1–26.8]	37.0% (34/92) [27.1–47.7]

Real-time PCR was performed on blood, urine and kidney. Seroprevalence was assessed with MAT (cut-off 1:40) for 26 serovars. Chi-squared independence test from contingency table was performed to determine difference of seroprevalence between stray and domestic cats and did not highlight a significant difference (*χ*^2^ (1, *N* = 92) = 0.77; *P* = 0.38). Fisher's exact test showed a significant difference in PCR results from urine samples (*P* = 0.038), but not form blood samples (*P* = 0.058), and could not be calculated for renal samples. CIs at 95% are indicated in brackets. *P < 0.1, **P < 0.05.

**Table 3.** Individual status for MAT and blood, urine and kidney tissue PCR for the twelve cats presenting at least one positive result at PCR analysis

Cat ID	Blood	Urine	Kidney	Serological results
E15	−	−	+	–
E19	−	NE	+	1:80 (19 Icterohaemorrhagiae)
				1:80 (COP Copenhageni)
				1:40 (VER Icterohaemorrhagiae)
E22	−	−	+	–
E25	−	+	−	–
E27	−	+	−	–
E29	+	−	−	1:40 (19 Icterohaemorrhagiae)
E31	+	−	−	–
E32	−	+	+	1:80 (19 Icterohaemorrhagiae)
				1:40 (COP Copenhageni)
E35	+	−	−	–
E37	−	−	+	1:40 (19 Icterohaemorrhagiae)
				1:40 (COP Copenhageni)
E41	+	+	+	–
E43	+	−	−	–

NE, non-evaluated; +, positive result for *L. interrogans* via qPCR and sequencing.

If the animal was seropositive, the seroprevalence titre is indicated for all serovars detected.

Cat ID, identification number associated with the animal for the study.

Based on *rrs* gene PCR and sequencing, *L. interrogans* species was identified in all positive stray cat samples.

When comparing the molecular results with the serological results, out of 92 cats, 4 (4%) were positive by both PCR and serological methods, 30 (33%) were seropositive but had negative PCR results and 8 (9%) were seronegative but had at least one positive PCR result. In total, 42/92 cats (46%) were positive by at least one of the tests performed.

Regarding the four animals with positive results via both MAT and PCR, all of them were seropositive for the serogroup Icterohaemorrhagiae. One PCR result was positive only in the blood sample, one was positive only in the renal sample, one was positive in both renal and urine samples and one was positive in the renal sample without PCR analysis of the urine sample (volume of urine too small for investigation).

## Discussion

In the present study, we evaluated the prevalence of *Leptospira* infection among cats on Reunion Island, in order to investigate the role of feline species as maintenance hosts of different strains of *Leptospira* spp. Serological (MAT) and molecular (PCR) methods were used in order to determine the infectious status of 92 cats, including 42 stray cats and 50 domestic cats. Sampling of blood (*n* = 92), urine (*n* = 89) and kidneys (*n* = 45) were performed in 2013. At that time, our study was the most important in terms of the number of cats included and tested on Reunion Island [25]. It was also the first study to investigate whether the living condition of cats (domestic *vs.* stray) influenced infection status or seroprevalence. We found no significant difference in seroprevalence between stray and domestic cats. However, our molecular results highlighted the presence of *L. interrogans* in the blood, kidneys and urine of stray cats, but not domestic cats, which should be considered in leptospirosis control policies.

In this study, we found 37% (cut-off 1:40) of seroprevalence for *Leptospira* infection, which is close to the only study published regarding the cat population in the island at that time that reported 26.6% (cut-off titre 1:100) [15]. When looking at studies performed in other countries published over the last 5 years, the mean seroprevalence observed in cats was 14%, with only one study with a prevalence over 20% in Serbia (cut-off titre 1:100) [11, 13, 16, 19, 26–31]. Our study records a high seroprevalence in cats, which could be due to a lower cut-off value (1:40). Moreover, knowing that there is currently no leptospirosis vaccine for cats due to the low morbidity of the disease, seropositivity is a true indicator of exposure and cannot be confused with postvaccination seropositivity. This high seropositivity suggests that cats from Reunion Island are in contact with the bacteria, probably through other maintenance host species such as rodents (predator-chain transmission), within a favourable environment for *Leptospira* spp. transmission (tropical climate) [17, 32].

None of the animals tested presented clinical signs (for domestic cats) or macroscopic lesions (for stray cats) compatible with leptospirosis (fever, weight loss, icterus, lethargy, ascites that could reflect renal failure, with hepatitis) [10]. This is consistent with the idea that cats are weakly susceptible to show clinical signs of *Leptospira* infection. Nevertheless, clinical cases of leptospirosis have been reported in cats infected with higher titres (at least one serovar above 1:800) of serovar Pomona (more pathogenic than the serovars we found in Reunion Island) [10]. In conclusion, cats do not seem to be clinically susceptible to the *Leptospira* serovars circulating in Reunion Island.

Cross-reactions between two or more serogroups represented 32% of positive MAT in our study. This confirms the difficulties encountered in identifying infecting serovars in patients and in maintenance hosts. However, serogroups Icterohaemorrhagiae, Ballum and Cynopteri were detected in a significant proportion of domestic cats, and serogroups Icterohaemorrhagiae and Australis were detected in a significant proportion of stray cats.

Interestingly all human epidemic surveys in Reunion Island reported *L. interrogans* serogroup Icterohaemorrhagiae to be the predominant infecting serogroup. Between 2004 and 2012, this serogroup represented 57.4% of the confirmed human cases [25]. Serogroup Australis has also been identified occasionally [33]. In animals, considered potential maintenance hosts or sensitive species, such as rats, cattle and dogs, *L. interrogans* species was also predominantly identified, with the occasional presence of *L. borgpetersenii* and *L. kirschneri* detected in a few animals [34]. In our study, the same species and serogroups were identified in cats, suggesting important inter-species contamination. This contamination could occur through predation of murine species, especially for stray animals.

To study the potential role of cats in shedding bacteria in the environment, PCR was performed on blood, urine and kidney samples. The results revealed leptospiral DNA in 13.0% of the animals tested, but in 28.6% of the animals when considering only the stray cat population. More specifically, kidney carriage of leptospiral DNA may represent a risk of contamination of the environment. In our study, the renal carriage was estimated at 13% (6/46) among sampled cats, which is lower than what was recorded in 2012 by Desvars *et al*. (28.6%) [15]. This difference may be due to different sampled feline species population (different areas, way of life and proximity to cattle), or difference of sample conservation throughout the experiments. Moreover, the high Ct (30–34) obtained for qPCR suggested that the DNA excretion is close to the detection limit, which could also lead to false-negative results. We detected pathogenic *Leptospira* DNA in urine of 10% (4/39) of stray cats (4.5% of all 89 sampled cats), which is more than what was already published (0% and 0.6% [15, 17]). Interestingly, in a study conducted recently, authors discovered a correlation between *rrs2* and *lipL32* DNA sequences from a case of *L. borgpetersenii* detected in a feral cat and the DNA previously sequenced in a human case of leptospirosis in Reunion Island [17]. Altogether, it suggests that contamination from cats to humans could be possible, which supports the hypothesis of cats as maintenance host population for *Leptospira* spp. [17]. Another hypothesis could be to consider cats as bridge hosts that link maintenance host population (mainly rats) and susceptible species of concern (mainly human, cattle and pets) [35]. Indeed, they present characteristics of maintenance host population but could harbour fewer bacteria in their kidney and urine than rats (since all positives samples showed a high cycle threshold (Ct) between 30 and 34, which means that small amount of DNA is detected).

As observed in previous studies [27, 36], the actual bacterial DNA carriage in seropositive animals is relatively low (11.8% in our study), confirming that MAT is probably not the best method to detect active potential maintenance host species for *Leptospira* infection. In this case, various scenarios can be described in relation to the status of the animals tested. First, 54% of the animals presented negative results in both MAT and PCR. Regarding the sensitivity of the methods involved, those animals represent the percentage of the population that has probably not been exposed to leptospires. Similarly, 33% of the animals were seropositive with no leptospiral DNA detected by PCR. This part of the population may contain the animals that have been exposed to the bacteria but have eliminated it with the initiation of the adaptive immune response; or did not excrete *Leptospira* at the time of sampling. Among the 8/92 animals (9%) presenting positive results in PCR and negative results in MAT, DNA was detected only in the blood for three of them, which is consistent with a recent exposure and ongoing infection with bacteraemia between the 3rd and 10th day post-exposure [7]. During this period, leptospires can invade renal tubules, and urinary excretion is possible for several months [37]. This is observed in cat number E41 with DNA detected in the blood, urine and kidneys. Subsequently, leptospires in the blood are eliminated by the immune system, and only renal carriage persists (animals E15 and E22) with intermittent urinary shedding. Interestingly, two cats were tested positive for DNA in urine sample but not in kidney sample (E25 and E27). This can be explained by the fact that DNA extraction was performed from small pieces of kidneys where bacteria may not have been present. Indeed, rodents and other animals asymptomatically carry leptospires in the lumen of proximal renal tubules [38, 39]. This also suggests that renal carriage recorded by PCR could be underestimated. Another aspect to consider is the potential impact of storage conditions on the quality of the DNA that could lead to an underestimation of prevalence. Finally, four cats presented positive results by both PCR and MAT with the Icterohaemorrhagiae serogroup. The blood sample from E29 was positive via PCR and presented a titre of 1:40, which could be characteristic of the initiation of adaptive immunity in an animal with relatively recent infection. This particular case could also be the result of a new infection with serological traces of a previous infection, as leptospiraemia is known to last only a few days [7]. In E36, leptospiral DNA was detected in the kidney sample, and the MAT titre was 1:40. This is indicative of an old infection and chronic carriage. E32 exhibited the same situation, but DNA was also detected in the urine, which is indicative of intermittent urinary excretion. Finally, for E19, leptospiral DNA was detected in the kidney sample, but PCR could not be performed with the urine sample from this animal.

The PCR results showed a significant difference between the two populations, with 12/42 positive results for stray cats compared with 0/50 in domestic cats. These data must be considered carefully for several reasons. First, because few of the domestic cats (3/50) were evaluated for renal samples. Consequently, some of those animals could carry leptospires in their renal tubules which may not have been detected in urine samples. Second, even if cats rarely present clinical signs of leptospirosis, clinical examinations were only performed on domestic cats, and this could represent a bias of population recruitment. The way the recruitment was performed aimed at limiting this bias by verifying the absence of lesions characteristic for leptospirosis during the necropsy of stray cats. Finally, the small sample size in this study hindered a robust comparison of the two groups. Despite those limitations, the PCR results led us to hypothesise that the environment of the animal could influence the actual chronic kidney carriage of the bacteria. Higher rates of excretion of *Leptospira* among stray cats could be linked with exposition to higher doses or more frequent exposure due to their environment, contact with stray dogs and predation on small mammals. In addition, their immune system could be compromised and less efficiently control the bacteria carriage due to lower general health or co-infections, with feline immunodeficiency virus for example [40].

Species identification analysis performed on positive samples confirmed that the cats from our study harbour a pathogenic species (*L. interrogans*). As previously reported [17], leptospiral DNA (*L. borgpetersenii*) detected in cats can be congruent with genome sequences reported for human cases. Interestingly, we show evidence of exposure to diverse serogroups, but evidence of infection with *L. interrogans* only. Even, if the serological and genomic classifications of *Leptospira* genus do not correlate, we suggest here that several serogroups associated with several different species of *Leptospira* circulate in the island with probably different maintenance communities. These observations highlight the idea of leptospirosis being a multi-host–multi-pathogen system with strong variability between different ecosystems. In this situation and with the significant carriage we reported in stray cats, controlling the population of stray animals on the island is an increasingly important public health issue as they very probably are part of the maintenance community for *L. interrogans*. Moreover, more public awareness campaigns should be undertaken to advise people in contact with stray animals about the risks of transmission.

Future investigations could be performed to improve data collection on cat populations. Positive samples by PCR could be grown on tween-albumin EMJH medium [21] to determine whether the detected DNA originates from dead or living leptospires. Indeed, several recent studies reported the isolation of pathogenic *Leptospira* spp. from kidneys and urine of cats [18, 19]. This information could provide crucial insights into the actual risk following urinary excretion. Regarding the MAT results, it would be interesting to perform a second serological measure after a 2-weeks’ time interval for the animals with bacteraemia to consider the sensitivity of MAT in asymptomatic and recently infected animals. Combining MAT and PCR analyses of blood, urine and kidney samples, our study confirms that cats are involved in the epidemiological cycle of leptospirosis, and that special care must still be taken with stray cat populations on Reunion Island. Nevertheless, we still have to determine if cats are contaminated through the predator-chain transmission with rats (being the main source of infection), if transmission within-species occurs between cats, if they are contaminated from the environment, or a combination of all, in various proportions.

## Data Availability

The data that support the findings of this study will be available on request via e-mail from the corresponding author.
